# Candidate genes involved in metastasis of colon cancer identified by integrated analysis

**DOI:** 10.1002/cam4.2071

**Published:** 2019-03-18

**Authors:** Yiming Zhou, Yiwen Zang, Yi Yang, Jianbin Xiang, Zongyou Chen

**Affiliations:** ^1^ Department of General Surgery Huashan Hospital, Fudan University Shanghai China

**Keywords:** bioinformatic analysis, colon cancer, metastasis, TCGA

## Abstract

Colon cancer is one of the most malignant cancers worldwide. Nearly 20% of all colon cancer patients are diagnosed at stage IV (metastasis). However, further study of colon cancer is difficult due to a lack of understanding of its pathogenesis. In this study, we acquired high–throughput sequence data from TCGA datasets and performed integrated bioinformatic analysis including differential gene expression analysis, gene ontology and KEGG pathways analysis, protein–protein analysis, survival analysis, and multivariate Cox proportional hazards regression analysis in order to identify a panel of key candidate genes involved in the metastasis of colon cancer. We then constructed a prognostic signature based on the expression of REG1B, TGM6, NTF4, PNMA5, and HOXC13 which could provide significant prognostic value for colon cancer.

## 1 INTRODUCTION

Colon cancer is one of the most malignant cancers worldwide. Unfortunately, nearly 20% of all colon cancer patients are diagnosed at stage IV (metastasis) every year.^1^ Many survival analyses have demonstrated that metastasis can sharply reduce the survival rate; for example, the 5‐year survival rate is 92% for stage I while it is only 11% for stage IV.^2^ In colon cancer treatment, 5‐flurouracil remains one of the most effective and commonly used drugs. However, up to 50% of metastatic colon cancers are chemo‐resistant to 5‐flurouracil, which is a pressing concern in clinical treatment.^3^ It is urgent that we solve the mystery of metastasis in colon cancer.

Many studies have focused on the mechanism of colon cancer as well as metastasis, but second–generation gene sequencing is the most promising in terms of uncovering the causes and pathogenesis of colon cancer as well as identifying novel biomarkers with great prognostic value.^4^

In this study, we performed integrated analysis including differential gene expression analysis, gene ontology analysis, KEGG pathway analysis, survival analysis, and multivariate Cox proportional hazards regression analysis to identify a panel of key candidate genes involved in the metastasis of colon cancer. Using these data, we constructed a prognostic signature based on the expression of REG1B, TGM6, NTF4, PNMA5, and HOXC13 which could provide great significant prognostic value for colon cancer. Compared to the TNM stage of colon cancer, the risk score system can be used as an independent factor in clinical prognosis.

## 2 MATERIALS AND METHODS

### 2.1 Data source

High–throughput sequence data and clinical data were acquired from the TCGA dataset, which included 480 colon cancer samples as of 15 May 2018. As TCGA is a publicly available dataset, no ethics approval was needed. We divided these patients according to N stage and M stage into two groups. Both N0 and M0 patients were included in the nonmetastasis group while all others were included in the metastasis group for the following analysis.

### 2.2 Differential gene expression in colon cancer

We determined differentially expressed genes using the edgeR package with a cutoff of *P* < 0.05 and|logFC|≥1.5.^5^ Heatmap was drawn by the pheatmap R package. To better explore the biological significance of DEGs, the clusterProfiler R package was utilized to conduct the gene ontology and KEGG pathway analysis,^6^ and significant enrichment was defined as *P* < 0.05. Since many of these genes interact with each other, the STRING database was applied to construct the interaction network of genes and determine the central genes.^7^ Cytoscape software was used to visualize the relationship between genes.^8^

### 2.3 Survival analysis to screen the candidate genes

Colon cancer samples were divided into two groups according to gene expression: high expression (with TPM values higher than the median), and low expression (with TPM values lower than the median). Then we used Kaplan‐Meier method to analyze the candidate genes of significant prognostic value with *P* < 0.05.

### 2.4 Construction of a prognostic signature on candidate genes

Multivariate Cox proportional hazards regression analysis was performed on the candidate genes to predict the risk score of colon cancer patients. The risk score for predicting overall survival was calculated as follows:

expRNA1×βRNA1+expRNA2×βRNA2+expRNA3×βRNA3+…expRNAn×βRNAn,

where expRNA is the expression level of RNA and βRNA is the regression coefficient calculated by the multivariate Cox proportional hazards regression model. Patients were divided according to the risk score into two groups: the high–risk group and the low–risk group. Then, KM‐plot survival analysis was performed between the two groups, and the Log–rank test was utilized to analyze the differences between groups. The predictive value score, including sensitivity and specificity of risk, was assessed by ROC analysis.

### 2.5 Prognostic value assessment

We regrouped the patients according to the clinical parameters, such as age, gender, BMI, location of tumor, histological type, TNM stage, neoplasm status, and risk level. Additionally, to evaluate the relationship between risk level and other clinical parameters, a chi‐square test was performed. A *P* < 0.05 was considered a significant correlation.

In clinical diagnosis and treatment, location of colon cancer and TNM stage are the major factors which influence prognosis. In this study, we aimed to assess the clinical application value of this risk score system. Thus, the clinical parameters with risk scores were analyzed in univariate Cox proportional hazards regression. Then, factors with a *P <* 0.05 were taken into multivariate Cox proportional hazards regression. In this step, an index with a *P* < 0.05 can be considered an independent prognostic factor. The results of the Cox regression model analysis were more clearly exhibited by the forestplot R package.

## 3 RESULTS

### 3.1 Differently expressed genes involved in colon cancer

**Figure 1:**
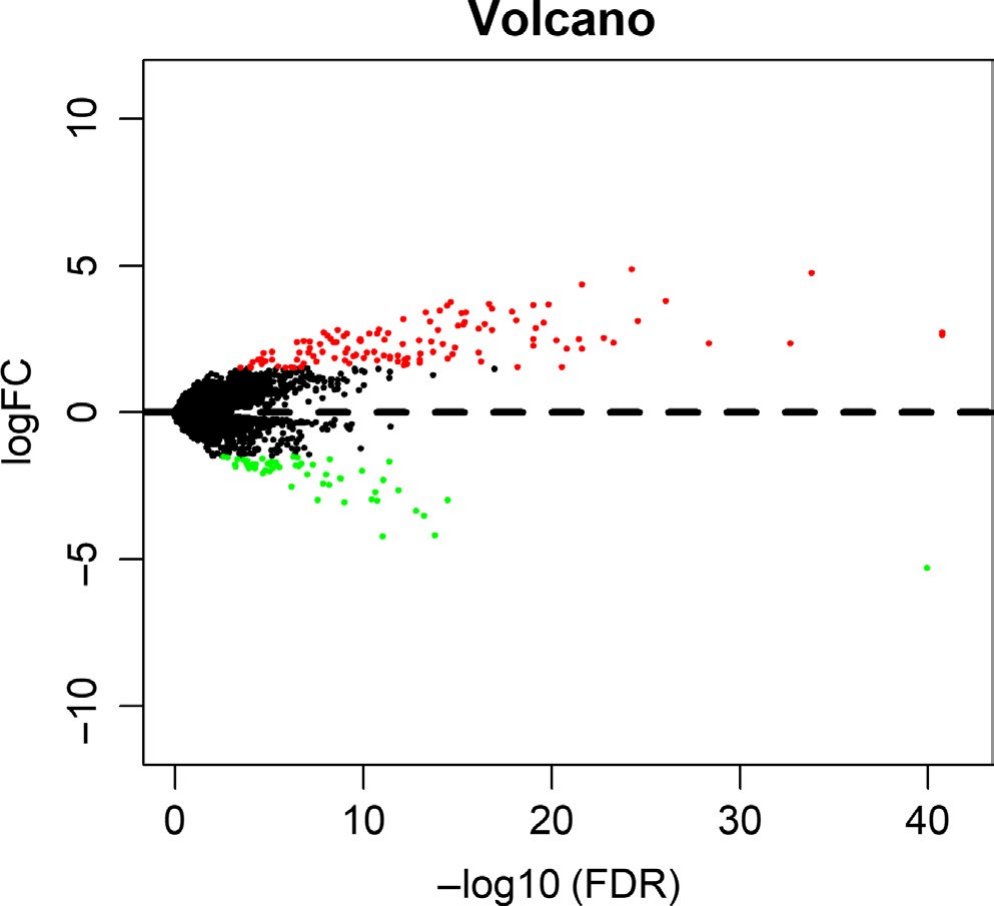
Volcano map for dysregulated genes involved in metastasis. The y‐axis value is logFC and the x‐axis value is −log10(FDR), the red plots mean up‐regulated while green plots mean down‐regulated

**Figure 2:**
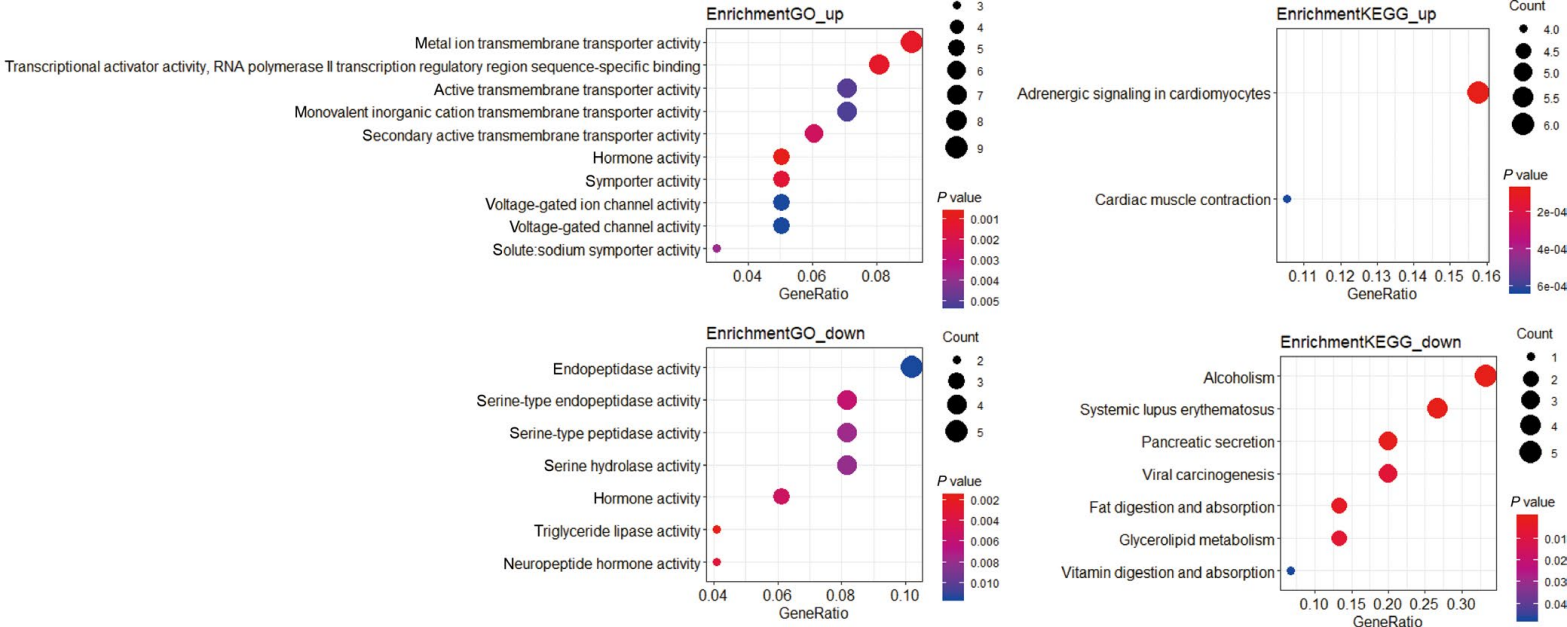
Enrichment analysis of dysregulated genes. Upper left panel shows the GO analysis of up‐regulated genes; lower left is the GO analysis of down‐regulated genes. Upper right panel is the KEGG analysis of up‐regulated genes; lower right panel shows the KEGG analysis of down‐regulated genes

**Figure 3:**
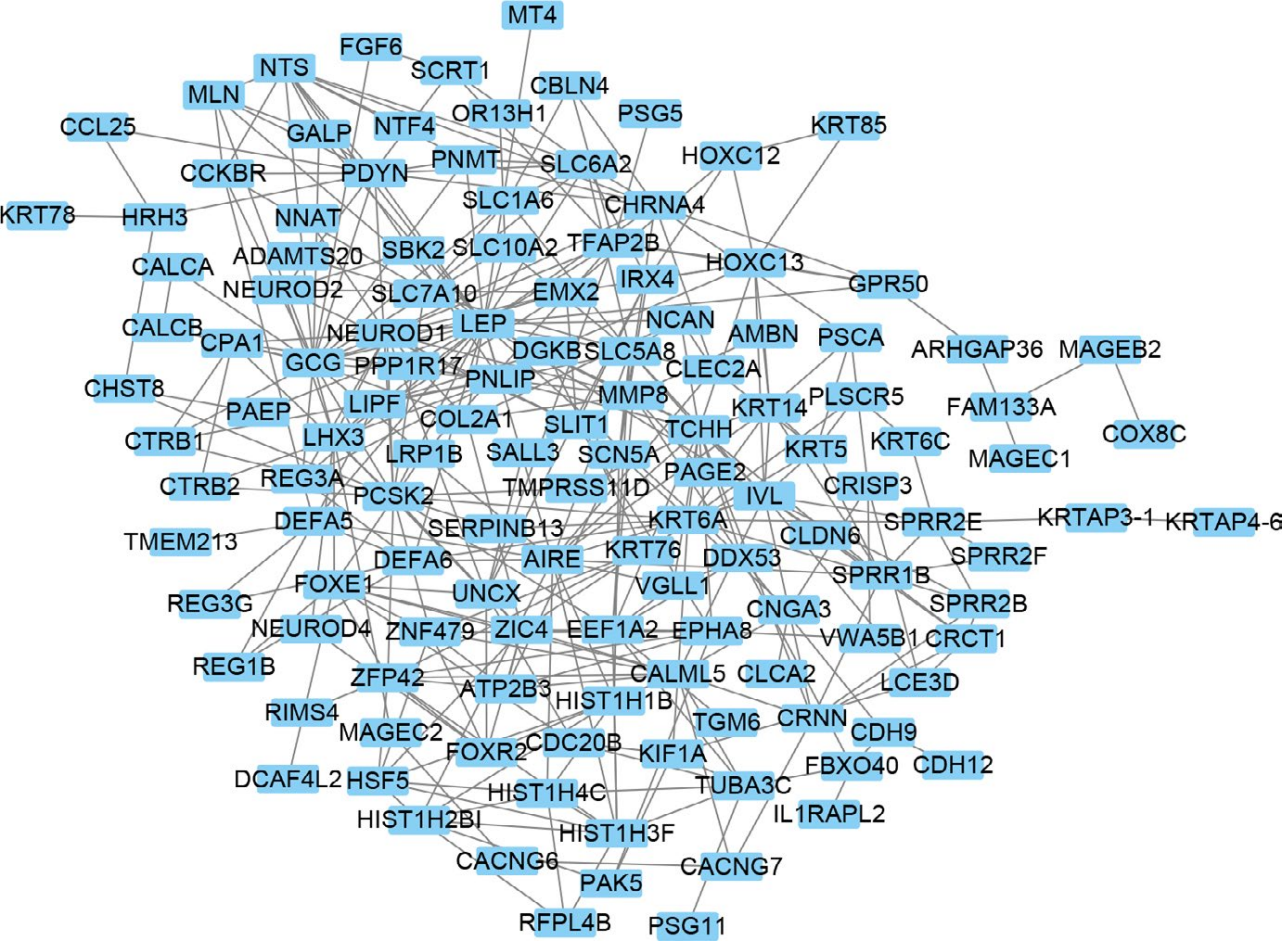
Protein–protein network of dysregulated genes. Each spot represents the protein while line between spots means the interactions

In colon cancer, both N0 and M0 patients were defined as the nonmetastasis group, while all others were defined as metastasis group. One hundred seventy‐three genes were dysregulated according to the cutoff of *P* < 0.05 and |logFC|≤1.5. Among them, 53 genes were down‐regulated and 120 genes were up‐regulated (Figure 1, Figure S1). To further evaluate the genes’ functions, we performed gene ontology analysis (GO) and Kyoto Encyclopedia of Genes and Genomes (KEGG) analysis. The up‐regulated genes are mainly enriched in hormone activity, metal ion transmembrane transporter activity, transcriptional activator activity, RNA polymerase II transcription regulatory region sequence–specific binding, and adrenergic signaling in cardiomyocytes. The down‐regulated genes were primarily enriched in hormone activity, triglyceride lipase activity, neuropeptide hormone activity and pancreatic secretion (Figure 2). We then used the STRING website to estimate the interactions between these genes. We used Cytoscape software to construct a network of 359 pairs involving 137 proteins (Figure 3).

### 3.2 Survival analysis of dysregulated genes

**Figure 4:**
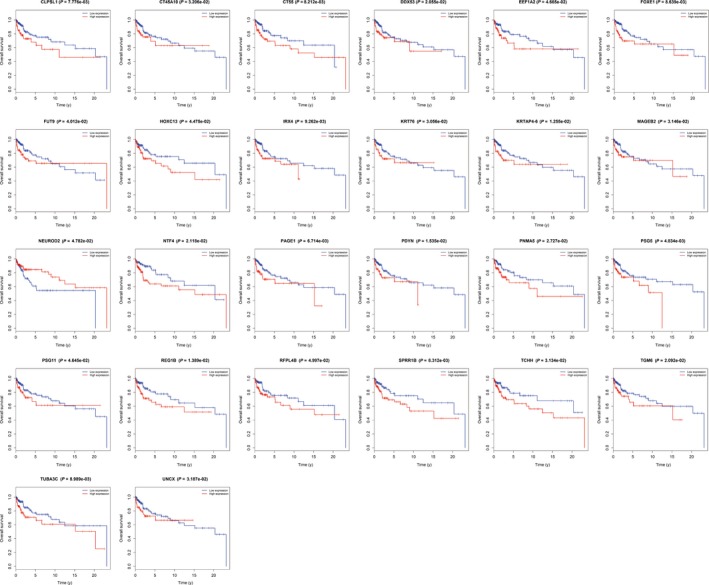
Survival analysis of dysregulated genes. Red line represents the high expression of patients while the blue line represents the low expression of patients

In colon cancer, patients in late stages have a poorer clinical outcome than patients in early stages. To find the candidate genes which may influence survival outcomes, we performed survival analysis on all dysregulated genes. Twenty‐six candidate genes were found to impact overall survival days: PSG5, PAGE1, CLPSL1, CT55, SPRR1B, FOXE1, TUBA3C, IRX4, KRTAP4‐6, REG1B, PDYN, DDX53, TGM6, NTF4, PNMA5, KRT76, TCHH, MAGEB2, UNCX, CT45A10, FUT9, HOXC13, PSG11, EEF1A2, NEUROD2, and RFPL4B, respectively (Figure 4).

### 3.3 Construction the prognostic signature based on candidate genes

We performed multivariate Cox proportional hazards regression analysis on the candidate genes to determine whether the signatures consisting of REG1B, TGM6, NTF4, PNMA5, and HOXC13 exhibited a significant prognostic value. The risk score for predicting the overall survival was calculated as follows:

Risk score=expREG1B×0.0832+expTGM6×0.1776+expNTF4×0.2406+expPNMA5×0.1281+expHOXC13×0.1226

**Figure 5:**
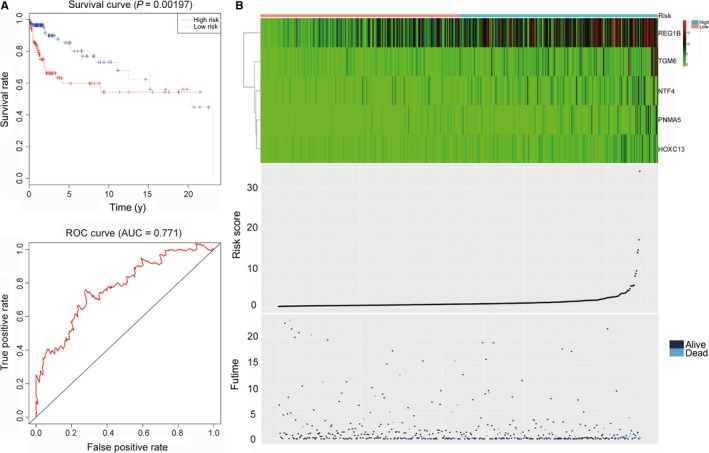
The prognostic signature in colon cancer. (A) The upper panel represents the survival analysis of the prognostic while the lower is ROC analysis. (B) The upper panel is a heatmap of five genes, the middle panel is the risk score of each colon cancer, and the lower panel is the survival status and overall survival time of each colon cancer

**Figure 6:**
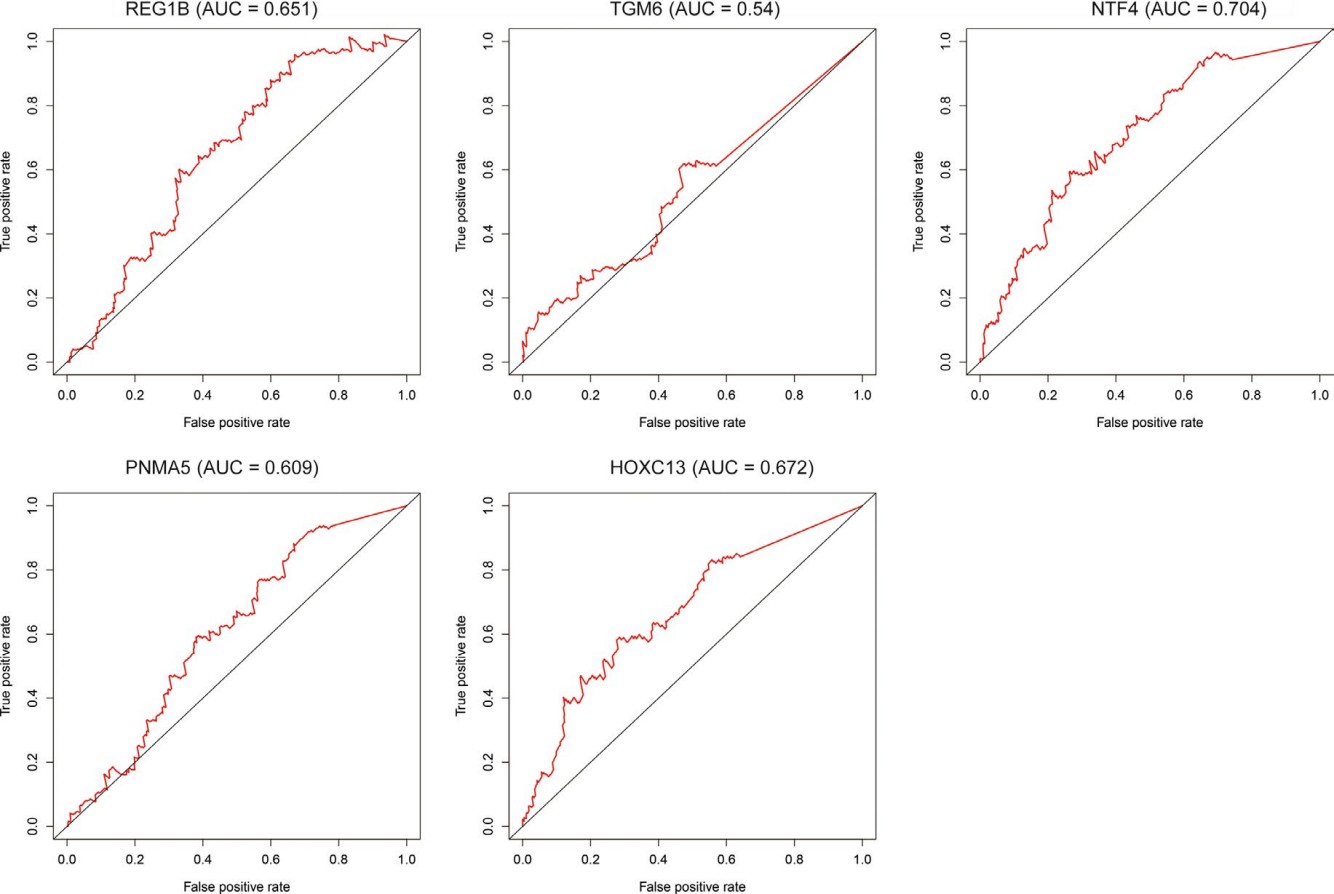
Receiver operating characteristic curve of 5 genes. ROC was performed for REG1B, TGM6, NTF4, PNMA5 and HOXC13 for the prognostic value in colorectal cancer

According to the risk score, colon cancers were divided into two groups: the high–risk group and the low–risk group. Survival analysis showed that a significant difference existed between the two groups (*P* < 0.001), with the high–risk group tending to have a poorer prognosis than the low–risk group. In the receiver operating characteristic (ROC) curve, the area under the curve (AUC) is 0.771, demonstrating that the risk score assessment is accurate (*P* < 0.05, 95% CI of HR:0.538‐0.703, sensitivity:0.704, specificity:0.496) (Figure 5). Furthermore, the prognostic value, including sensitivity and specificity of these five genes, were performed by ROC analysis (Figure 6).

### 3.4 Prognostic value assessment

As shown in Table 1, clinical parameters consisting of age, gender, BMI, location of carcinoma, TNM stage, histological type, neoplasm status and risk level were divided into two groups. In a chi‐square test, risk level has a significant correlation with tumor location (*P* = 0.009), TNM stage (*P* = 0.049) and neoplasm status (*P* < 0.001) (Table 2).

**Table 1:** clinical parameters of colon cancer patients

Subgroup	Frequency	Percent	Valid percent
Age			
<60	121	27.4	27.4
≥60	320	72.6	72.6
Gender			
Male	231	52.4	52.4
Female	210	47.6	47.6
BMI			
<25	74	16.8	33.2
≥25	149	33.8	66.8
Location			
Left	174	39.5	45.2
Right	211	47.8	54.8
Histology			
Adenocarcinoma	365	85	85.8
Mucinous adenocarcinoma	62	14.1	14.2
Stage			
I + II	249	56.5	56.5
III+IV	192	43.5	43.5
T stage			
Tis,T1‐3	387	87.8	87.8
T4	54	12.2	12.2
N stage			
N0	258	58.5	58.5
N1 + N2	183	41.5	41.5
M stage			
M0	328	74.4	83.7
M1	64	14.5	16.3
Neoplasm status			
Tumor free	190	43.1	48.6
With tumor	201	45.6	51.4
Vital status			
Alive	387	87.8	87.8
Dead	54	12.2	12.2
Risk level			
Low	221	50.1	50.1
High	220	49.9	49.9

**Table 2:** Relationship between risk level and clinical parameters

Subgroup	High risk	Low risk	Total	*P*
Age				0.352
<60	56 (12.70%)	65 (14.74%)	121	
≥60	164 (37.19%)	156 (35.37%)	320	
Gender				0.318
Male	110 (24.94%)	121 (27.44%)	231	
Female	110 (24.94%)	100 (22.68%)	210	
BMI				0.058
<25	23 (10.31%)	51 (22.87%)	74	
≥25	66 (29.60%)	83 (37.22%)	149	
Location				0.009^*^
Left	74 (19.22%)	100 (25.97%)	174	
Right	118 (30.65%)	93 (24.16%)	211	
Histology				0.177
Adenocarcinoma	183 (41.88%)	192 (43.94%)	375	
Mucinous adenocarcinoma	36 (8.24%)	26 (5.94%)	62	
Stage				0.049^*^
I + II	114 (25.85%)	135 (30.61%)	249	
III+IV	106 (24.04%)	86 (19.50%)	192	
T stage				0.238
Tis, T1‐3	189 (42.86%)	198 (44.90%)	387	
T4	31 (7.03%)	23 (5.21%)	54	
N stage				0.195
N0	122 (27.66%)	136 (30.84%)	258	
N1+N2	98 (22.22%)	85 (19.28%)	183	
M stage				0.435
M0	167 (42.60%)	161 (41.07%)	328	
M1	36 (9.18%)	28 (7.15%)	64	
Neoplasm status				<0.001^*^
Tumor free	72 (18.41%)	118 (30.18%)	190	
With tumor	120 (30.69%)	81 (20.72%)	201	
Vital status				0.078
Alive	187 (42.40%)	200 (45.35%)	387	
Dead	33 (7.49%)	21 (4.76%)	54	

**Figure 7:**
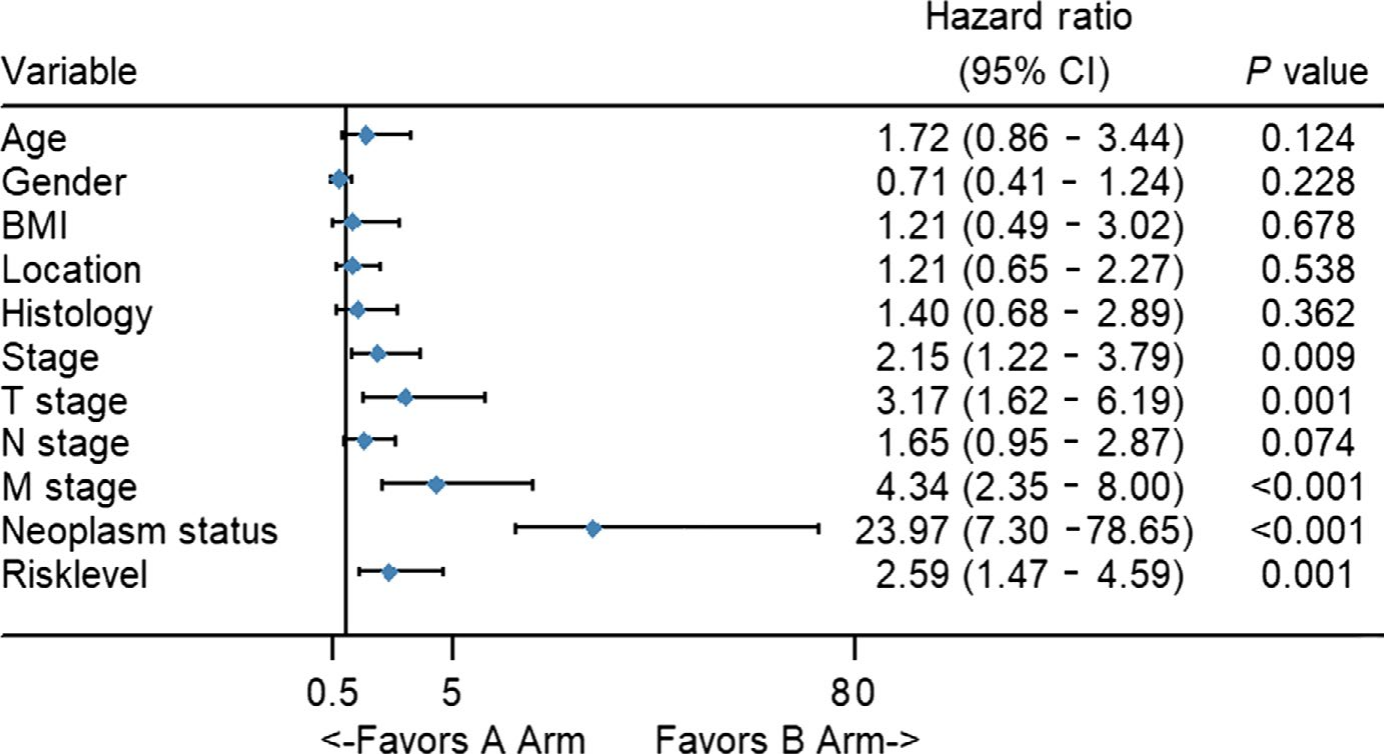
Forest map of univariate logistic regression analysis. The line shows the 95% CI, and the location of the diamond on the line represents the odds ratio

**Figure 8:**
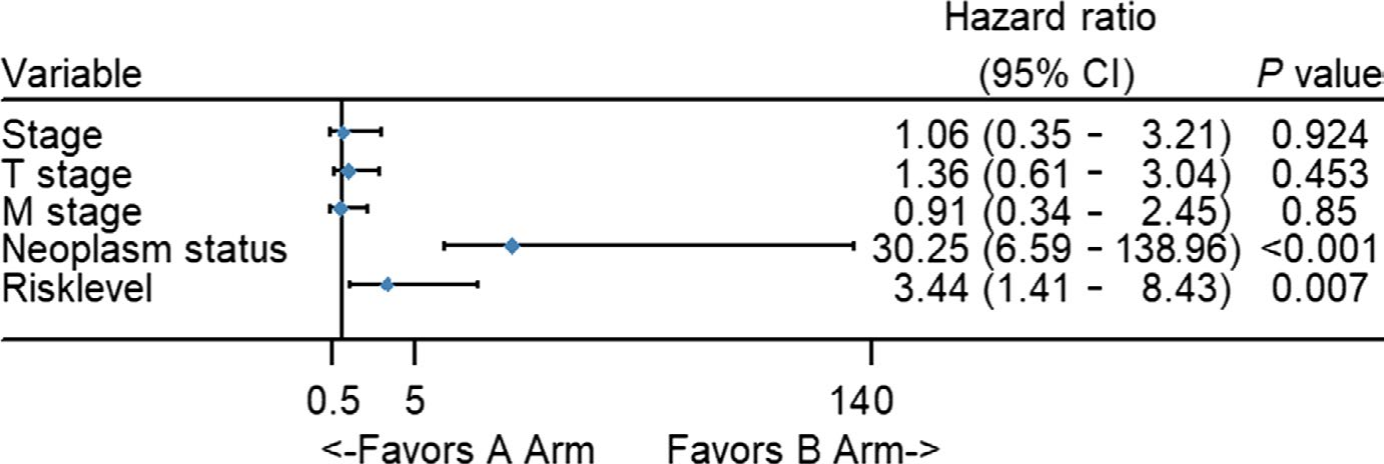
Forest map of multivariate logistic regression analysis. The line shows the 95% CI, and the location of the diamond on the line represents the odds ratio

To compare the prognostic power of the risk score system with clinical parameters, univariate Cox proportional hazards regression analysis was performed. We found that TNM stage, T stage, M stage, neoplasm and risk level are indicators of poor outcomes. (Figure 7). Additionally, these five indexes were entered into multivariate Cox proportional hazards regression analysis. Unexpectedly, neoplasm and risk level both had a *P <* 0.05, indicating that they can be utilized as independent factors in evaluating clinical outcomes. TNM stage, T stage, and M stage can be used as prognostic factors but cannot be used independently (Figure 8).

## 4 DISCUSSION

Colorectal cancer (CRC) is a heterogeneous disease with different mechanisms of pathogenesis, and it is important to explore the molecular biomarkers for early diagnosis, prognosis prediction, and metastasis diagnosis. Past studies that have utilized deep sequencing and proteomics assays reported varied biomarkers for colorectal cancer diagnosis. Such varied biomarkers were found because of the limited number of tumor specimens and the range of genomic backgrounds of patients. One of these biomarkers, CDX2, is a homeobox gene encoding transcriptional factors for intestinal organogenesis and has been confirmed to be a strong prognostic factor in stage II and III CRC.^9^ Additionally, peroxisome proliferator–activated receptor gamma coactivator 1 alpha (PGC‐1α) can be used as a biomarker of physical activity–protective effect on colorectal cancer.^10^ MUC2 was also identified as a biomarker; low levels of MUC2 in CRC tissues predict a poor outcome independent of stage or other well‐–recognized markers of later–stage disease. However, large, well–designed cohort studies are required to validate MUC2 as a biomarker for poor prognosis in CRC.^11^ High circulating IL‐6 was associated with short overall survival in most studies in CRC cancer patients and may be used as a therapeutic target of CRC.^12^ Furthermore, classic biomarkers, including RAS, BRAF, and microsatellite instability (MSI), can also be used for CRC diagnosis.^13^ Though various biomarkers have been explored, no ideal biomarker was identified for clinical application. Thus, further research on biomarkers was necessary.

In this study, we performed several bioinformatics analyses to identify key genes involved in metastasis of colon cancer. At first, we found that 173 genes were dysregulated between nonmetastatic and metastatic colon cancer. Among them, 26 candidate genes impact overall survival. The prognostic signature based on REG1B, TGM6, NTF4, PNMA5, and HOXC13 could divide colon cancer patients into high–risk and low–risk groups, with an AUC of the ROC of 0.771.

REG1B is a member of the REG family, which is composed of antiapoptotic factors and growth factors that affect epithelial cells within the digestive system. The protein encoded by REG1B is highly similar to REG1A, which is also a member of the REG family. Studies showed that REG1A is an independent predictor of poor outcomes in patients with gastric cancer, breast cancer and colon cancer.^14^, ^15^, ^16^ Also, REG1A protein promoted cell growth and cell resistance to H_2_O_2_–induced apoptosis in AGS cells.^17^ In bladder cancer, decreased REG1A expression suppresses growth, invasion and angiogenesis.^18^ Upregulation of REG1A accelerates tumor progression in pancreatic cancer both in vitro and in vivo.^19^ As for REG1B, a study showed that both the expression of REGIα and REGIβ are up‐regulated in human β cells under inflammatory conditions through the JAK/STAT pathway.^20^ Furthermore, since REG1B is overexpressed in pancreatic cancer, the panel of CA199, SYCN and REG1B shows promise as an improved diagnostic indicator of pancreatic cancer.^21^ REG1B is also up‐regulated in colon cancer, and it could inhibit HCT116 proliferation, invasion, and cell cycle.^22^

TGM6 encodes an enzyme that catalyzes the cross‐linking of proteins and the conjugation of polyamines to proteins. Several studies showed that TGM6 is involved in acute myeloid leukemia and nervous system development, although its function in solid tumors remains to be elucidated.^23^, ^24^. However, TGM3, an important paralog of TGM6, has been studied in cancer. In esophageal squamous cell carcinoma, TGM3 could regulate cell proliferation, and the prognostic value of it was higher than those of the lymph node metastasis, intramural metastasis and vascular invasion status.^25^, ^26^, ^27^

NTF4 is a member of a family of neurotrophic factors, which have demonstrated multifunctional roles both in central and peripheral nervous system and perhaps serve as axonal guidance molecules during the growth and regeneration of nerves.^28^ It has also been determined that they stimulate axonal growth by mediating the polymerization and accumulation of F‐actin in growth cones and axon shafts. Furthermore, in fetal ovaries, NTF4 could potentially promote human follicular assembly.^29^ Because prostate cancer tends to invade neural tissue, NTF4 has also been studied in and stained ductal cells.^30^ NTF4 could stimulate cell proliferation and migration through its receptor both in melanoma and O‐2A cells.^31^, ^32^

PNMA5, a member of paraneoplastic Ma family which has been identified as containing onconeuronal antigens, has not been widely studied so far. In HeLa and MCF‐7, the c‐terminus of PNMA5 is required for nuclear targeting and localization, and it could promote apoptosis and chemo‐sensitivity.^33^

Homeobox–containing genes constitute a family of transcription factors that are highly conserved through evolution. In humans, there are 39 HOX genes spread throughout four different clusters: A, B, C and D. Homeobox–containing genes are a family of transcription factors regulating normal development and controlling primary cellular processes including cell identity, cell division and differentiation. HOXC13 is overexpressed in metastatic melanoma tissues compared to primary melanoma tissues and is targeted by miR‐503 in esophageal squamous cell carcinoma. It has been shown that HOXC13 promotes proliferation and inhibits apoptosis of ESCC.^34^. Furthermore, NUP98/HOXC13 is of pathogenetic importance in acute myeloid leukemia.^35^

In the survival analysis of this study, clinical parameters including age, gender, BMI, location of cancer, histology, TNM stage, T stage, N stage, M stage, and neoplasm cancer were analyzed along with risk level. Surprisingly, there were no significant differences regarding location of cancer on the left or right side, but this may be due to the limited sample size. Furthermore, in this survival analysis, we did not consider the impact of the patient's economic status, health insurance coverage, or treatment method (surgery, chemotherapy, radiotherapy, neoadjuvant therapy), all of which significantly influence clinical outcomes.

In clinical diagnosis and treatment, colon cancer status is typically determined through imaging or abnormally elevated serum tumor markers such as CEA, AFP, CA125, CA199. Generally, TNM stage and neoplasm cancer are used in identifying high risk patients, but these are difficult to utilize in clinical treatment without a quantitative index. In this study, we performed integrated analysis to discover the differentially expressed genes involved in metastasis of colon cancer and also to construct a prognostic signature based on the candidate genes to better evaluate the outcome of colon cancer patients. We found that the prognostic signatures consisting of REG1B, TGM6, NTF4, PNMA5, and HOXC13 exhibited a significant prognostic value. Furthermore, since expression of these genes is a marker of poor prognosis in patients with colon cancer, these may be useful therapeutic targets for precision medicine. Further studies are still needed to explore the biological functions and underlying molecular mechanisms of REG1B, TGM6, NTF4, PNMA5, and HOXC13 in colon cancer.

## CONFLICT INTEREST

The authors declare that they have no competing interests.
