# Supplementary figures and images for: Candidate genes involved in metastasis of colon cancer identified by integrated analysis
Source: Cancer Med. 2019 Mar 18;8(5):2338–47. doi: 10.1002/cam4.2071 (PMC6536975; doi:10.1002/cam4.2071)

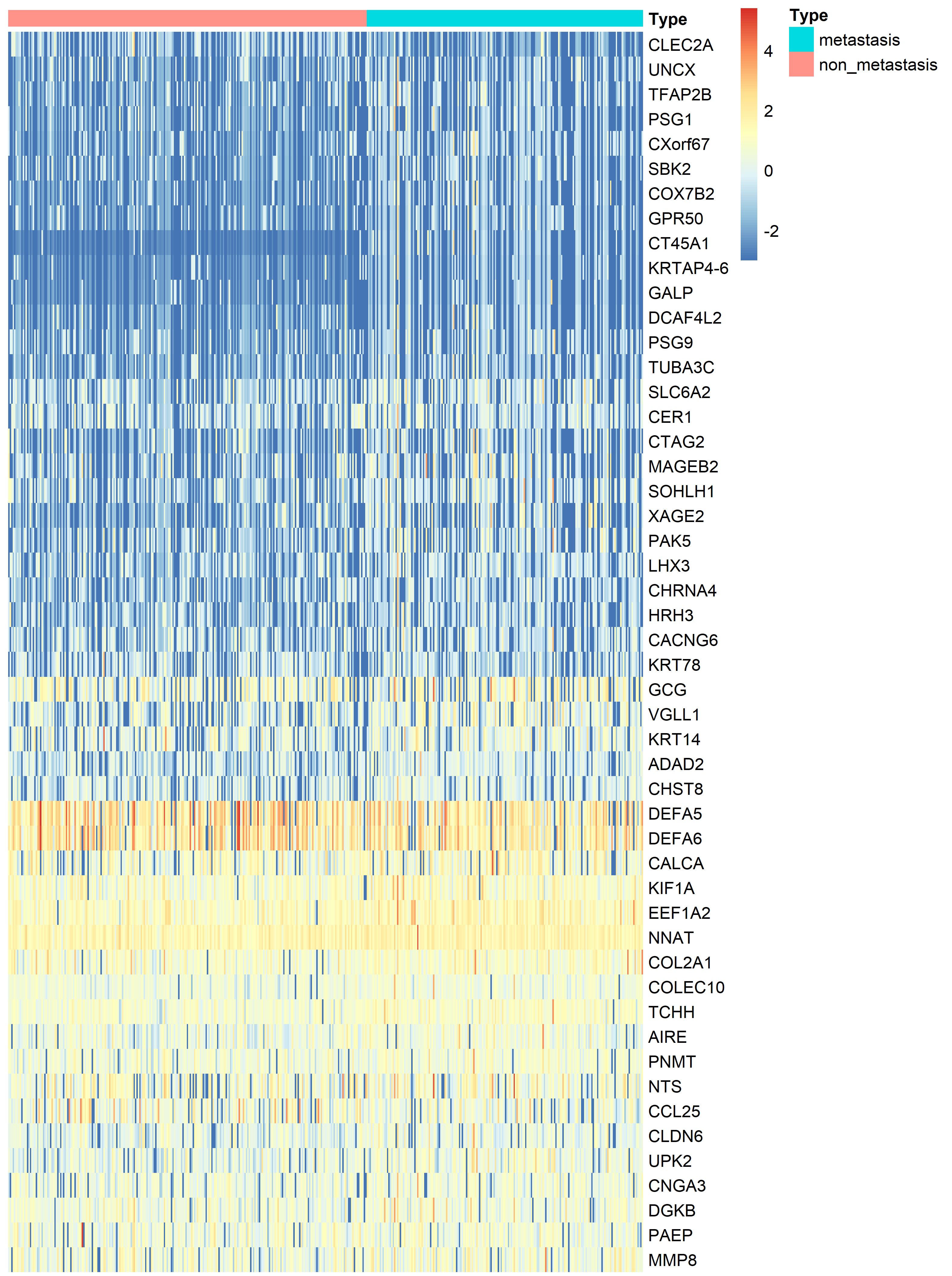

Supplement: Supplementary file 1 [file CAM4-8-2338-s001.tiff]
